# Automatic removal of soft tissue from 3D dental photo scans; an important step in automating future forensic odontology identification

**DOI:** 10.1038/s41598-024-63198-2

**Published:** 2024-05-30

**Authors:** Anika Kofod Petersen, Andrew Forgie, Dorthe Arenholt Bindslev, Palle Villesen, Line Staun Larsen

**Affiliations:** 1 Department of Forensic Medicine, Aarhus University, Aarhus, Denmark; 2 School of Medicine, Dentistry and Nursing, University of Glasgow, Glasgow, Scotland; 3 Department of Dentistry and Oral Health, Aarhus University, Aarhus, Denmark; 4 Bioinformatics Research Centre, Aarhus University, Aarhus, Denmark; 5 Department of Clinical Medicine, Aarhus University, Aarhus, Denmark

**Keywords:** Forensic odontology, Identification, Data science, Automated comparison, 3D scans, IOS (intraoral scanner), Data processing, Forensic dentistry

## Abstract

The potential of intraoral 3D photo scans in forensic odontology identification remains largely unexplored, even though the high degree of detail could allow automated comparison of *ante mortem* and *post mortem* dentitions. Differences in soft tissue conditions between *ante-* and *post mortem* intraoral 3D photo scans may cause ambiguous variation, burdening the potential automation of the matching process and underlining the need for limiting inclusion of soft tissue in dental comparison. The soft tissue removal must be able to handle dental arches with missing teeth, and intraoral 3D photo scans not originating from plaster models. To address these challenges, we have developed the grid-cutting method. The method is customisable, allowing fine-grained analysis using a small grid size and adaptation of how much of the soft tissues are excluded from the cropped dental scan. When tested on 66 dental scans, the grid-cutting method was able to limit the amount of soft tissue without removing any teeth in 63/66 dental scans. The remaining 3 dental scans had partly erupted third molars (wisdom teeth) which were removed by the grid-cutting method. Overall, the grid-cutting method represents an important step towards automating the matching process in forensic odontology identification using intraoral 3D photo scans.

## Introduction

Identifying casualties after a disaster is a critical task^1^. Forensic odontology identification, which is one of the primary identification methods according to Interpol^2^, involves forensic odontologists comparing *ante mortem* dental records with *post mortem* dental findings^1–3^. Unlike the other primary identifiers (fingerprints and DNA), dentitions can provide information about the estimated age and lifestyle of the victim, thus providing a dental profile of the individual even when identification is not possible at this stage^4^. Dentitions are made up of the most resilient material of the human body and dentitions are well protected in the oral cavity and therefore less likely to deteriorate and more likely to withstand trauma^4^. Identification of individuals with little to no dental work is difficult as the dental record generally contains sparse data with little to no identifying traits in these cases^2,3,5^. These cases most often end up with the conclusion *identification probable, identification possible*, or *insufficient evidence*, especially in disasters with many victims^4^. 3D photo scanning is increasingly used in dentistry and although individuals may have no previous dental work done, their dental records may contain intraoral 3D photo scans (dental scans); e.g., recorded for orthodontic screening and treatment planning^6^. Currently, 3D photo scans are not fully implemented in ordinary disaster victim identification^7^. Dental scans create detailed 3D recordings of the dental surfaces, allowing computational analysis of dentitions by using the 3D tooth shapes^6,8–10^. For future forensic dental comparison it is recommended to use the short distance tooth shapes, as inaccuracies have been reported on the long-distance measures^10–13^. Performing *post mortem* dental scans could facilitate an initial, automated comparison of tooth shapes with a database of *ante mortem* dental scans as shown by Reesu et al.^9,10^. This potential automation could streamline a part of the matching process within forensic odontology identification in the future^9^.

Surrounding soft tissue in both *ante mortem* and *post mortem* scans may interfere with accurate comparison because soft tissue can add unwanted variation between scans, without contributing significant discriminative information^14,15^. Therefore, the amount of soft tissue included in the dental scans before automated comparison should be limited. One approach is to remove soft tissue from the dental scan using the acquisition software, which requires manual data cleaning for each dental scan. Especially in cases with many victims, manual removal of soft tissue from *ante mortem* dental scans would require a significant investment of human resources. A few studies have reported on automated soft tissue removal from 3D dental scans^15–19^. The methods of these studies aimed at determining the precise border between dentition and soft tissue but some of the methods suffer from poor performance on dentitions with missing teeth, malocclusion or dental scans not originating from plaster models ^15,16^. The use of supervised deep learning for tooth segmentation has also been emerging ^15,17–19^. For supervised deep learning, the lack of representation of dentitions with multiple missing teeth in the training data poses a concern for their performance in a forensic setting ^15,17–19^. The aim of the present study was to develop a new robust method for automated soft tissue removal from dental scans that works with scans from both living and deceased humans with or without missing teeth and malocclusion. Unlike earlier methods focusing on defining the dentition/soft tissue border ^15,16^, our method aims at limiting the amount of soft tissue in dental scans. Our study mimics real-life disaster victim identification scenarios since the goal is to automate data cleaning to support future automated forensic odontology identification.

## Materials

The study was conducted in full accordance with ethical principles, including the World Medical Association Declaration of Helsinki. Further, the study was registered with the Data Protection Unit at Aarhus University, Denmark (file number 2016–051-000001, serial number 2534 and file number 20220367531, serial number 3155) and complied with the European Union General Data Protection Regulation legislation. The chairmanship of The Danish National Committee on Health Research Ethics (NVK) has assessed that the study and its protocol is exempt from notification (case number: 2400741) and the study is thus approved. The study was conducted in two parts. Part A was a pilot study with in vivo scans of six dental arches from three consenting donors from the donation programme at Department of Biomedicine, Aarhus University, with missing teeth and gaps in the dental arches. Since the new robust method successfully removed soft tissue from all six dental arches without removing teeth, a larger study (B) was conducted. Part B used sixty dental arch scans from thirty healthy volunteers after giving written informed consent (12 self identified males, 18 self identified females, ages 23–61 years). Seven of the 60 dental arches in part B had at least one missing tooth (not including dental arches with missing third molars (wisdom teeth)) and all the dental arches were scanned in vivo.

**Figure 1:**
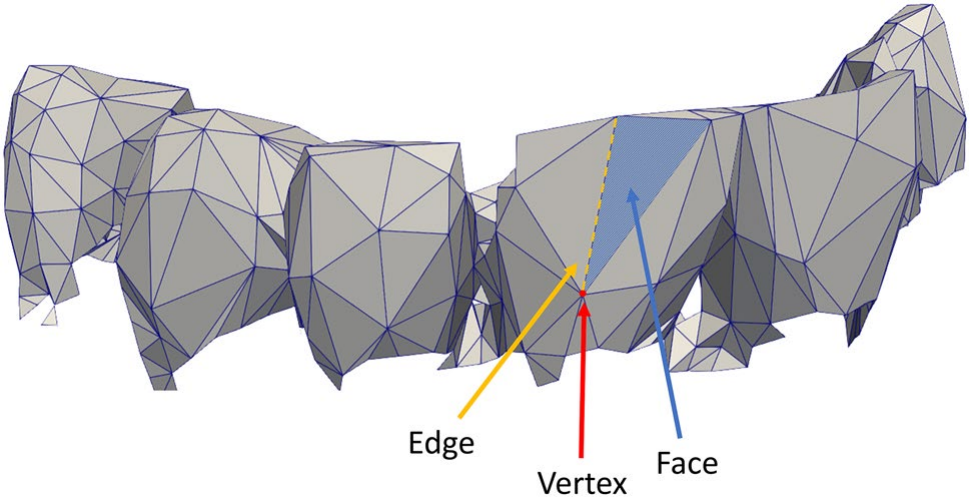
Nomenclature of meshes. Simplified mesh showing a vertex, an edge, and a face.

The scanning was performed by a forensic odontologist or by trained personel using an intraoral 3D photoscanner [Primescan AC, Sirona Dental Systems GmbH, Germany]. The dental scans were not subject to any prior removal of soft tissue. The dental scans were stored in STL-files as meshes using vertices (points in 3D space), edges between vertices, and faces (a plane defined by its edges) (Fig. 1), with unit size of the cartesian coordinate system of 1 mm.

## Methods

In short, the grid-cutting method applies a grid to the dental scan in the xy-direction, whereafter each grid square is cut independently with a plane to remove soft tissue from the teeth of the dental scan. The grid-cutting method can be divided into four distinct steps. The first step entails approximating the occlusal plane through the use of algorithm 1 (supplementary, algorithm 1). The second step involves dividing the dental arch into contiguous squares by means of a grid. Subsequently, each square is analyzed individually in the third step, using algorithm 2 (supplementary, algorithm 2), in order to determine how much of the original dental scan that needs to be removed. Finally, the three steps are combined into a single function using algorithm 3 (supplementary, algorithm 3).

The grid-cutting method requires only three flexible user variables: the grid (*G*) size, the inclusion criterion (*I*) and the ratio of inclusion (*R*). *G* specifies the degree of granularity when dividing the grid. *I* determines how much of the mesh within each square to exclude in the resultant cropped dental scan. *R* dictates which grid squares are included in the final resultant cropped dental scan.

### Part one: occlusal plane approximation

The orientation of the dental scan is determined by the user's specifications regarding whether it is a maxillary or mandibular dental scan. The dental scan is automatically oriented with the occlusal surfaces/incisal edges in the positive z direction (up), effectively flipping the upper jaw upside down, to ensure equal processing of maxillary and mandibular dental scans.

**Figure 2:**
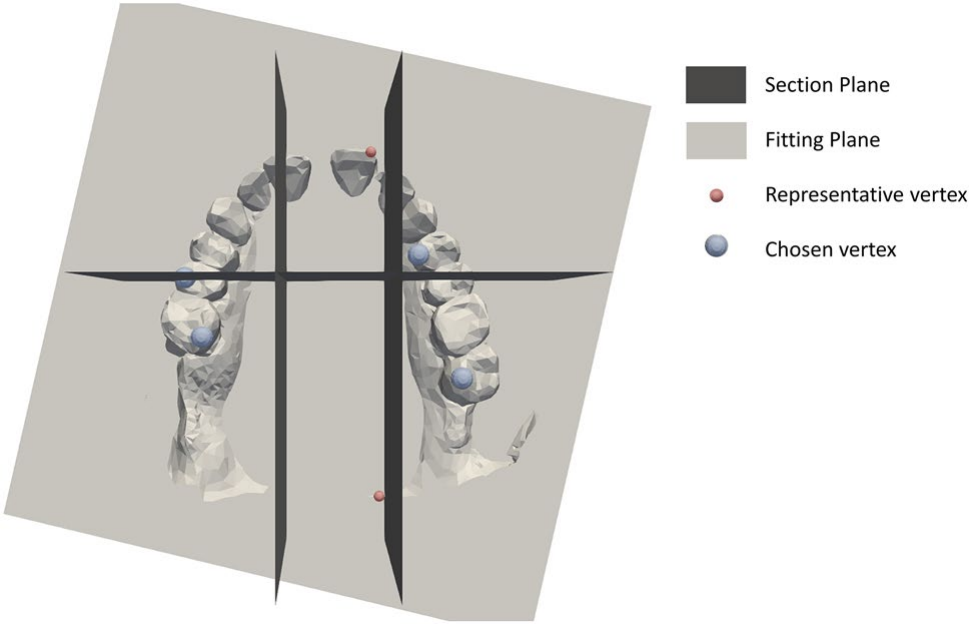
Plane sectioning and section representatives. To approximate the occlusal plane, the dental arch was split into 6 sections by 3 planes. For each section, a section representative vertex was found according to largest distance to the fitting plane. The four section representative vertices with the largest z-coordinate were chosen to estimate the approximated occlusal plane.

Algorithm 1 approximates the occlusal plane of the dental arch. To do so, algorithm 1 removes all vertices and faces below a fitting plane found using Mean Squared Error (MSE). The remaining vertices and faces are split into six sections, dictated by one plane in the y-direction, and two planes in the x-direction (Fig. 2). For each of the sections, a section representative vertex is chosen, to serve as an approximate occlusal point of that section. The section representative vertices are the vertex in each section with the largest distance to the fitting plane. Of the six section representative vertices, the four with the largest z-coordinate are chosen to estimate the occlusal plane. The occlusal plane is estimated using MSE on the four chosen points (Fig. 2).

### Part two: grid splitting

**Figure 3:**
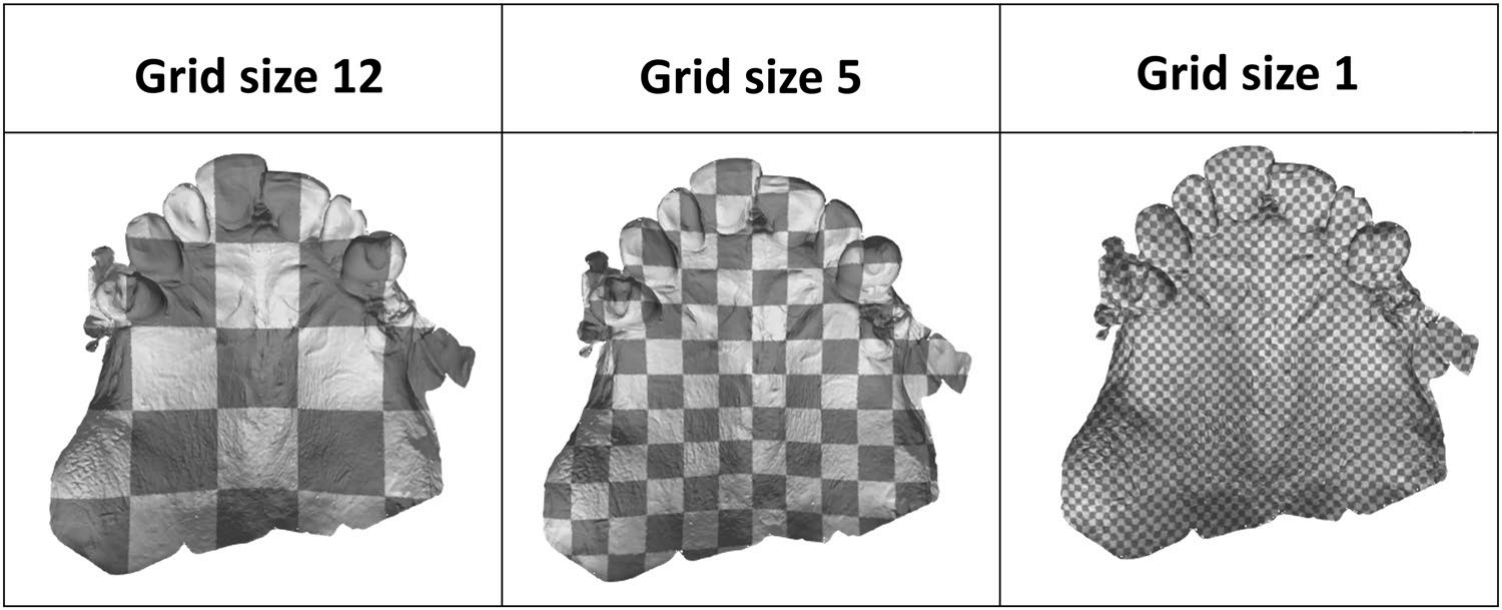
The effect of grid size. A smaller grid size causes a more fine-grained grid, since each square becomes smaller, and consequently, the number of squares increases.

Once the occlusal plane has been estimated, the dental scan is divided into squares of a user-defined size. These squares are created in the xy-direction (Fig. 3) and are analysed individually in part three.

### Part three: square analysis

Algorithm 2, which independently analyses each grid square, focuses on the vertex with the highest z-value. This vertex is referred to as the evaluation point. To expedite the computation of each grid square and enhance the speed of the algorithm, the evaluation point of the grid square is used, rather than the vertex closest to the approximated occlusal plane. To quickly eliminate squares with no teeth, the distance between the evaluation point and the approximated occlusal plane is compared to the total z-range of the scan. If the distance is larger than the ratio of inclusion (*R*) (default 33%, empirically determined) of the total z-range (the highest point in the square is far below the approximated occlusal plane), all vertices within that grid square are excluded from the cropped dental scan. If the z-value threshold is met, an inclusion threshold is established for that particular grid square. This inclusion threshold is defined by Eq. (1).

1 $$Inclusion\,\, threshold = E_{p} + I*N_{o}$$

where $E_{p}$ is the evaluation point of the grid square, *I* is the user defined inclusion criterion and $N_{o}$ is the normal vector of the approximated occlusal plane. The inclusion threshold is located *I* units (mm) from the evaluation point in the direction of the approximated occlusal plane normal vector. Any vertex with a z-value higher than the inclusion threshold will be included in the cropped dental scan. In other words, vertices closer to the approximated occlusal plane than a given threshold will be kept.

### Part four: method collection

Algorithm 3 integrates all previous stages into a unified method. Initially, the occlusal plane is estimated, followed by dividing the dental scan using a grid and analysing each grid square independently. Finally, vertices identified for removal during the square analysis are eliminated, and the cropped dental scan returned.

### Code availability

The unified method can be found at https://github.com/AnikaKofodPetersen/Grid-Cutting.

### Visualisation

Visualisation of dentitions was made using ParaView^20^.

## Results

**Figure 4:**
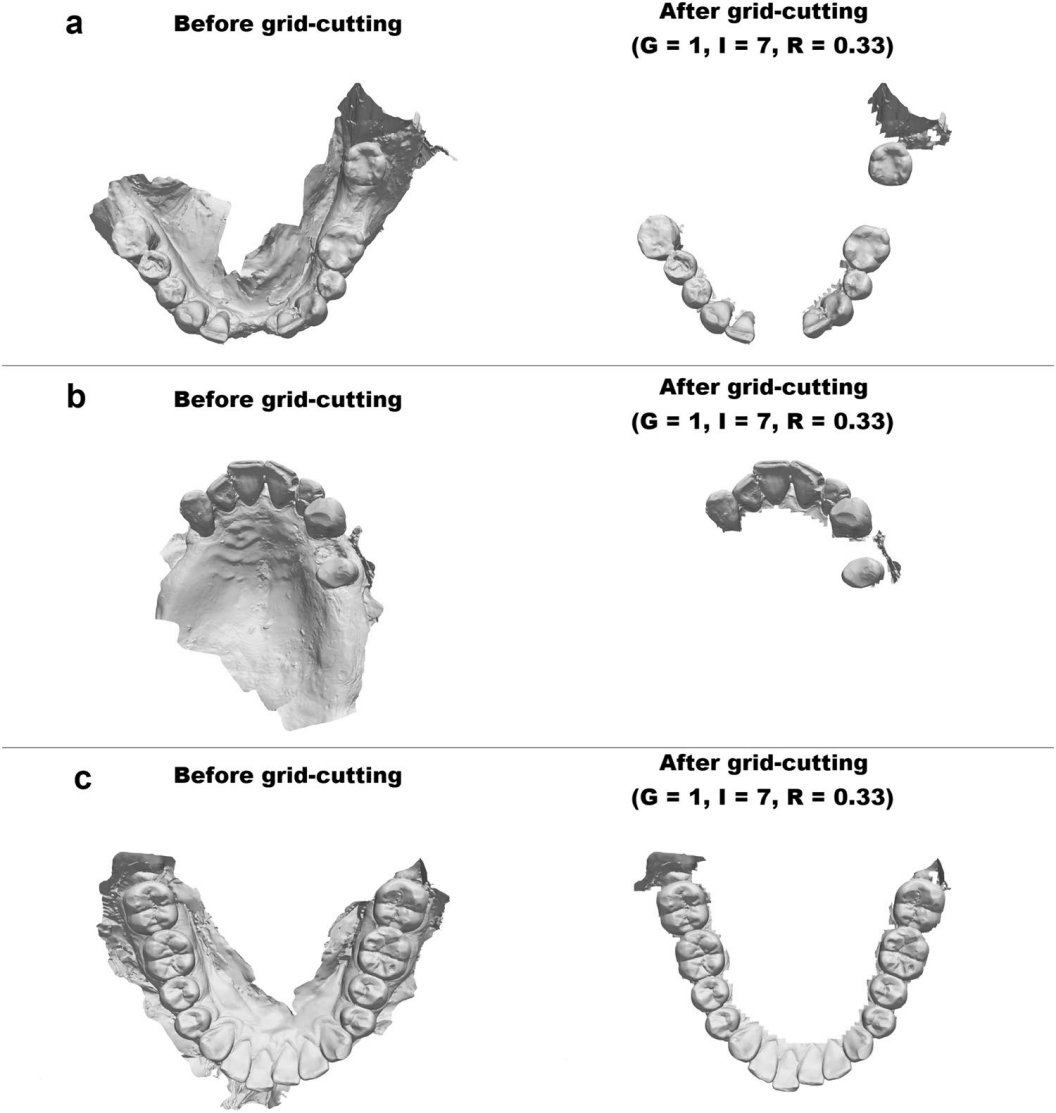
Grid-cutting on 3 dental arches. Grid-cutting was performed with grid size = 1, inclusion criterion = 7 and ratio of inclusion = 0.33 on all three dental arches.

Since study A showed no unwanted removal of dental surfaces, the findings from study A and study B are jointly presented. All dental arches were subject to grid-cutting (*G* = 1, *I* = 7, *R* = 0.33). The grid-cutting method successfully removed soft tissue on 58/66 dental arches without removing teeth. Three examples are shown in Fig. 4.

**Figure 5:**
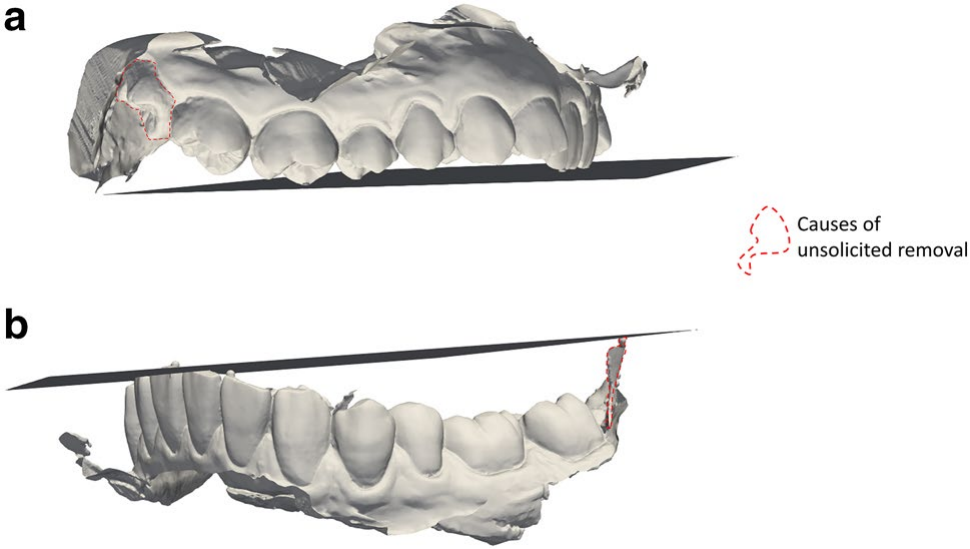
Examples of causes of unsolicited removal of grid squares. Depicted in relation to the approximated occlusal plane. (**a**) Ectopic and partly erupted third molar (wisdom tooth). (**b**) Retromolar area distorting the approximated occlusal plane.

The soft tissue remaining on the dental scans after grid-cutting was either a) the tissue in immediate proximity to the teeth (the gingiva) or b) part of a cheek, lip, or the area behind the last molar (retromolar area). Eight dental arches suffered removal of at least one grid square from one or more teeth. Five of these cases comprised removal of one or more partly erupted/ectopic third molars (wisdom teeth) positioned relatively far from the approximated occlusal plane (Fig. 5a). The three remaining cases of removal was due to inclusion of the retromolar area on one side, causing the approximated occlusal plane to be inaccurate (Fig. 5b).

**Figure 6:**
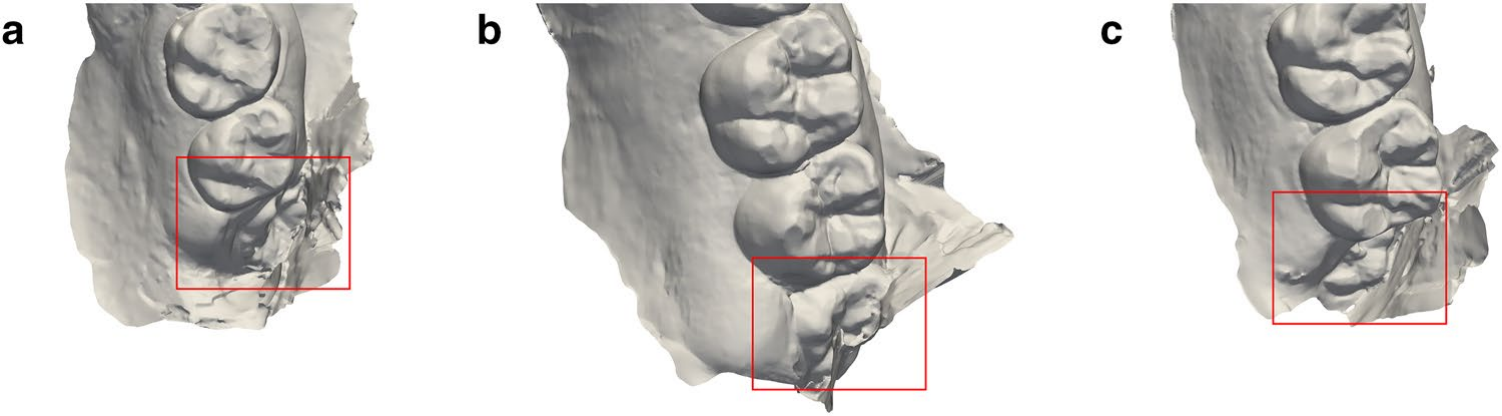
Examples of third molars (wisdom teeth) which were subject to unsolicited removal. Specific dental arches each with a partly erupted third molar (wisdom tooth). In these three cases, grid-cutting removed the tooth as if it was soft tissue due to its location compared to the approximated occlusal plane.

The extent of dental surface preservation depends on the user defined variables, inclusion criterion (*I*), grid size (*G*) and ratio of inclusion (*R*). *I* affects how far from the occlusal surfaces and incisal edges the grid-cutting method removes tissue. *G* dictates the area to be analysed in each grid square. Thus, *G* dictates the granularity of the method. *R* is used to evaluate whether the content of a grid square should be included in the final model. Consequently, the grid-cutting method's effectiveness in preserving dental surfaces depends on the user-defined variable *I, G* and *R*. The eight dental arches which had unwanted partial removal of teeth were resubjected to grid-cutting (*I* = 5, *G* = 5, *R* = 0.4), resulting in unsolicited removal of a third molar (wisdom tooth) in only three dental arches. In these specific cases, the tooth in question was only partly erupted and disclosed less than half of the dental crown (Fig. 6).

## Discussion

The grid-cutting method was tested on 66 dental arches, successfully removing soft tissue from 58/66 dental arches without removing teeth. The eight dental scans which suffered from unsolicited removal of one or more grid squares within teeth were characterized by either the inclusion of the retromolar area on one side, causing the approximated occlusal plane to be inaccurate, or the position of a partly erupted third molar (wisdom tooth) in relation to the approximated occlusal plane. These shortcomings were overcome in 5/8 dental arches by tweaking the user defined variables *I, G* and *R*. This is an improvement to the previously published methods ^15,16^, as the significance of maintaining teeth surpasses that of removing all soft tissue. The grid-cutting method shows robustness compared to previously published methods ^16^, as the grid-cutting method was able to handle data acquired orally (not originating from plaster models), as would be the case in disaster victim identification. Since the aim of the grid-cutting method is to limit soft tissue without removing teeth, the grid-cutting method does not accurately trace the teeth in order ensure complete removal of soft tissue. By adjusting the user-defined variables (inclusion criterion, grid size and ratio of inclusion), this method can be tailored to suit specific-use cases. If loss of teeth in certain cases is of no issue, the grid-cutting method can be tweaked, using the user defined variables, to remove more tissue, including dental tissue. This would be beneficial in cases where only occlusal surfaces and incisal edges are of particular interest. Currently forensic odontology identification mainly includes comparing *ante mortem* 2D dental record data with *post mortem* dentitions^1–3,7^. Prospectively, the degree of detail of intraoral 3D photo scans should be utilised in the identification process^6–8^, given that the unwanted variation caused by the soft tissue is limited^14,15^. Unwanted variation can be caused by notable differences in the soft tissue condition between dental scans, or due to difference in scanning techniques resulting in different levels of soft tissue included in the dental scans^8^. The grid-cutting method presented in this study successfully removes soft tissue from dental scans, showing the potential of automating parts of the forensic odontology process. To assess the practicability of this new promising method in future 3D-based forensic odontology identification, the next step will be to compare manual soft tissue removal with the grid-cutting method.

### Supplementary Information

Supplementary Information.

## Data Availability

The data could be shared after publication upon reasonable request via communication with the corresponding author.
