# Supplementary material for: Automatic removal of soft tissue from 3D dental photo scans; an important step in automating future forensic odontology identification
Source: Sci Rep. 2024 May 30;14:12421. doi: 10.1038/s41598-024-63198-2 (PMC11139984; doi:10.1038/s41598-024-63198-2)
Supplement: Supplementary file 1 — Supplementary Information. [file 41598_2024_63198_MOESM1_ESM.pdf]

Automatic removal of soft tissue from 3D dental photo scans; an important step in automating future forensic odontology identification

Authors

Anika Kofod Petersen[1*], Andrew Forgie[2], Dorthe Arenholt Bindslev[1,3], Palle Villesen[4,5], Line Staun Larsen[1,3]

Affiliations

[1] Department of Forensic Medicine, Aarhus University, Denmark [2] School of Medicine, Dentistry and Nursing, University of Glasgow, Scotland [3] Department of Dentistry and Oral Health, Aarhus University, Denmark [4] Bioinformatics Research Centre, Aarhus University, Denmark [5] Department of Clinical Medicine, Aarhus University, Denmark *email: anko@forens.au.dk

Supplementary

Algorithm 1: Approximating the Occlusal Plane

Input: Decimated mesh containing vertices and faces, M_{deci} = (Vertices V_{deci} = ($v_1, v_2, \ldots, v_n$), Faces F_{deci} = ($f_1, f_2, \ldots, f_n$))

# Fit a plane through all vertices, V, using MSE
fit_plane = fitplane(V_{deci})

# Limit mesh, M, to only include vertices and faces on the dental side of the fitted plane
$M_{limited}$ = cutWithPlane(M_{deci}, fit_plane)

# *Find planes to split the dental scan into 6 sections*
planeY = Plane(pos=centerOfMass($M_{limited}$)+offset1, normal = (1,0,0)

planeX1 = Plane(pos=centerOfMass($M_{limited}$)+offset2, normal = (0,1,0)

planeX2 = Plane(pos=centerOfMass($M_{limited}$)+offset3, normal = (0,1,0)

Use the planes to cut the mesh into sections
Q1, Q2, Q3, Q4, Q5, Q6 = cutWithPlane(M_limited, (planeY, planeX1, planeX2))

# *Find the point from each section with the largest distance to the fitted plane*
q1 = largestDistance(V_{Q1}, fit_plane)

q2 = largestDistance(V_{Q2}, fit_plane)

q3 = largestDistance(V_{Q3}, fit_plane)

q4 = largestDistance(V_{Q4}, fit_plane)

q5 = largestDistance(V_{Q5}, fit_plane)

q6 = largestDistance(V_{Q6}, fit_plane)

Collect four occlusal plane representative points with largest distance to the fit plane
P1, P2, P3, P4 = sort(q1, q2, q3, q4) [:4]

Output: Occlusal plane representative points P1, P2, P3

Algorithm 2: Analysis of Grid Square

Input: Point indexes PI = ($pi_1, pi_2, ..., pi_n$), Points P = ($p_1, p_2, ..., p_n$), Occlusal plane OP = (p0 = *(x,y,z),* $\vec{N}$ = *(a,b,c)*), Distance threshold D, Inclusion threshold I.

# *Collecting z-coordinates of points P*
z_coord = P[:,2]

# *Find point with highest z-coordinate*
top_point = P[argmax(z_coord)]

```
# Find distance from the top point to the occlusal plane OP
dist = distance(top_point, OP)

# Check if all points are within the distance threshold, D, of the occlusal plane OP
if dist < D:

    # Set inclusion threshold for z-coordinate according to the top point
    inclusion_thres = (top_point+I*N⃗)[2]

    # Find points, P, with z-coordinates smaller than the inclusion threshold
    z_coord_idxs = where((z_coord < inclusion_thres))

    # Define point indexes, PI, to be deleted from the final mesh
    del_idxs = PI[z_coord_idxs]
```

Output: Indexes of points from the original mesh that is to be deleted, del_idx

---

Algorithm 3: Approximate Removal of Soft Tissue

Input: Mesh containing vertices and faces, M = (Vertices V = ($v_1, v_2, ..., v_n$), Faces F = ($f_1, f_2, ..., f_n$)), size of grid square sides S, Inclusion threshold I, Rate of inclusion R.

```
# Copy and decimate mesh to approximately 5000 vertices for occlusal plane retrieval
M_deci = decimateMesh(M, num_vertices=5000)
P1, P2, P3 = occlusalPlane(M_deci)
occlusal_plane =  fitPlane([P1, P2, P3])

# Define plane normal vector to point in the negative z-direction
if occlusal_plane.normal_vector[2] >0:
    occlusal_plane.normal_vector = occlusal_plane.normal_vector*(-1)

# Find bounding box of full resolution mesh in format [xmin, xmax, ymin, ymax, zmin, zmax]
bounding_box = boundingBox(M)

# Define grid based on the size of the grid square sides, S.
 x_range = range(bounding_box [0]-S, bounding_box [1]+S, step_size = S)
 y_range = range(bounding_box [2]-S, bounding_box [3]+S, step_size = S)
```

```
# Define how far from the top point should be considered as dentition (25% of the range of z values)
z_bounds = bounding_box [5]- bounding_box [4]
dist_thres = R*z_bounds

#  Initialize empty collection of indexes to be deleted
delete_idx = [ ]

# Iterate through grid squares
for i in enumerate(x_range[:-1]):
    for j in enumerate(y_range[:-1]):

        # Collect axis ranges for current grid square
        x_interval = [x_range[i], x_range[i+1]]
        y_interval = [y_range[j], y_range[j+1]]

        #  Collect vertices and their indexes within the square x- and y -intervals
        square_idx = squareFilter(M, x_interval, y_interval)
        square_vertices = V[square_idx]

        # Only work with squares that contain vertices
        if len(square_idx) > 0:

            # Collect indexes of vertices that are to be removed
            remove_idx = analyseSquare(square_idx, square_vertices, occlusal_plane, dist_thres, I)
            delete_idx.append(remove_idx)

# Delete vertices from all grid squares simultaneously
$M_{dentition}$ = deleteVertices(M, delete_idx)

# Delete vertices that form small floating islands
$M_{dentition}$ = deleteIslands($M_{dentition}$)
```

Output: Cropped mesh, $M_{dentition}$, after approximate removal of soft tissue
